# Monotonicity, convexity, and inequalities for the generalized elliptic integrals

**DOI:** 10.1186/s13660-017-1556-z

**Published:** 2017-11-09

**Authors:** Tiren Huang, Shenyang Tan, Xiaohui Zhang

**Affiliations:** 1 Department of Mathematics, Zhejiang Sci-Tech University, Hangzhou, 310018 China; 2 Taizhou Institute of Sci. & Tech., NUST, Taizhou, 225300 China

**Keywords:** 33C05, 33E05, 26D20, generalized elliptic integrals of the first and second kinds, Gaussian hypergeometric function, monotonicity, convexity, inequality

## Abstract

We provide the monotonicity and convexity properties and sharp bounds for the generalized elliptic integrals $\mathscr{K}_{a}(r)$ and $\mathscr {E}_{a}(r)$ depending on a parameter $a\in(0,1)$, which contains an earlier result in the particular case $a=1/2$.

## Introduction

For real numbers *a*, *b*, and *c* with $c\neq0,-1,-2,\ldots $ , the Gaussian hypergeometric function is defined by

1.1 $$\begin{aligned} F(a,b;c;x)= {}_{2}F_{1}(a,b;c;x)=\sum _{n=0}^{\infty}\frac {(a,n)(b,n)}{(c,n)}\frac{x^{n}}{n!} \end{aligned}$$

for $x\in(-1,1)$, where $(a,n)$ denotes the shifted factorial function $(a,n)\equiv a(a+1)\cdots(a+n-1)$, $n=1,2,\ldots $ , and $(a,0)=1$ for $a\neq0$. It is well known that the function $F(a, b; c; x)$ has many important applications in geometric function theory, theory of mean values, and several other contexts, and many classes of elementary functions and special functions in mathematical physics are particular or limiting cases of this function [1–10].

In what follows, we suppose $r\in(0,1)$, $a\in(0,1)$, and $r'=\sqrt {1-r^{2}}$. The generalized elliptic integrals of the first and second kinds are defined as

1.2 $$\begin{aligned} &\mathscr{K}_{a}(r)=\frac{\pi}{2}F \bigl(a,1-a;1;r^{2}\bigr),\qquad\mathscr {K}'_{a}(r)= \mathscr{K}_{a}\bigl(r'\bigr), \end{aligned}$$

1.3 $$\begin{aligned} &\mathscr{E}_{a}(r)=\frac{\pi}{2}F\bigl(a-1,1-a;1;r^{2} \bigr),\qquad\mathscr {E}'_{a}(r)=\mathscr{E}_{a} \bigl(r'\bigr). \end{aligned}$$

In the particular case $a=1/2$, the generalized elliptic integrals $\mathscr{K}_{a}(r)$ and $\mathscr{E}_{a}(r)$ reduce to the complete elliptic integrals $\mathscr{K}(r)$ and $\mathscr{E}(r)$, respectively. Recently, the Gaussian hypergeometric function and generalized elliptic integrals have been the subject of intensive research [2, 3, 5, 8, 11–30].

Anderson, Qiu, and Vamanamurthy [31] considered the monotonicity and convexity of the function

$$\begin{aligned} f(r)=\frac{\mathscr{E}(r)-r^{\prime 2}\mathscr{K}(r)}{r^{2}}\cdot\frac {r^{\prime 2}}{\mathscr{E'}(r)-r^{2}\mathscr{K'}(r)}. \end{aligned}$$

One of the main results of [31] is the following theorem.

### Theorem 1.1

*The function* $f(r)$ *is increasing and convex from* $(0,1)$ *onto* $(\pi /4,4/\pi)$. *In particular*,

1.4 $$\begin{aligned} \frac{\pi}{4}< f(r)< \frac{\pi}{4}+ \biggl(\frac{4}{\pi}- \frac{\pi }{4} \biggr)r \end{aligned}$$

*for* $r\in(0,1)$. *Both inequalities given in* (1.4) *are sharp as* $r\rightarrow0$, *whereas the second inequality is also sharp as* $r\rightarrow1$.

Alzer and Richards [32] studied the corresponding properties of the additive counterpart

$$\begin{aligned} \Delta(r)&=\frac{\mathscr{E}(r)-r^{\prime 2}\mathscr{K}(r)}{r^{2}}-\frac {\mathscr{E'}(r)-r^{2}\mathscr{K'}(r)}{r^{\prime 2}} \end{aligned}$$

and obtained the following theorem.

### Theorem 1.2

*The function* $\Delta(r)$ *is strictly increasing and strictly convex from* $(0,1)$ *onto* $(\pi/4-1,1-\pi/4)$. *Moreover*, *for all* $r\in(0,1)$, *we have*

1.5 $$\begin{aligned} \frac{\pi}{4}-1+\alpha r< \Delta(r)< \frac{\pi}{4}-1+\beta r \end{aligned}$$

*with the best constants* $\alpha=0$ *and* $\beta=2-\frac{\pi}{2}$.

It is natural to extend Theorems 1.1 and 1.2 to the generalized elliptic integrals $\mathscr{K}_{a}(r)$ and $\mathscr{E}_{a}(r)$. In this paper, we show the monotonicity and convexity of the functions

1.6 $$\begin{aligned} f_{a}(r)=\frac{\mathscr{E}_{a}(r)-r^{\prime 2}\mathscr{K}_{a}(r)}{r^{2}}\cdot\frac {r^{\prime 2}}{\mathscr{E'}_{a}(r)-r^{2}\mathscr{K'}_{a}(r)} \end{aligned}$$

and

1.7 $$\begin{aligned} g_{a}(r)=\frac{\mathscr{E}_{a}(r)-r^{\prime 2}\mathscr{K}_{a}(r)}{r^{2}}-\frac {\mathscr{E'}_{a}(r)-r^{2}\mathscr{K'}_{a}(r)}{r^{\prime 2}}. \end{aligned}$$

Moreover, we obtain sharp inequalities for them. If $a=1/2$, then our results return to Theorems 1.1 and 1.2, which are contained in [31] and [32].

## Preliminaries and lemmas

In this section, we give several formulas and lemmas to establish our main results stated in Section 1. First, let us recall some known results for $F(a,b;c;x)$.

The following formulas for the hypergeometric function can be found in the literature [33–35]:

2.1 $$\begin{aligned} F(a,b;a+b+1;x)=(1-x)F(a+1,b+1;a+b+1;x), \end{aligned}$$

the differential formula

2.2 $$\begin{aligned} \frac{dF(a,b;c;x)}{dx}=\frac{ab}{c}F(a+1,b+1;c+1;x), \end{aligned}$$

the asymptotic limit

2.3 $$\begin{aligned} \lim_{x\rightarrow1^{-}}F(a,b;c;x)=\frac{\Gamma(c)\Gamma(c-a-b)}{\Gamma (c-a)\Gamma(c-b)},\quad c>a+b, \end{aligned}$$

and the contiguous relation

2.4 $$\begin{aligned} (\sigma-\rho)F(\alpha,\rho;\sigma+1;z)=\sigma F(\alpha,\rho;\sigma ;z)-\rho F(\alpha,\rho+1;\sigma+1;z), \end{aligned}$$

where $\Gamma(x)$ is the Euler gamma function.

### Lemma 2.1

([2], Lemma 5.2)

*Let* $a\in(0,1]$. *Then the function* $[\mathscr{E}_{a}(r)-r^{\prime 2}\mathscr {K}_{a}(r)]/r^{2}$ *is increasing and convex from* $(0,1)$ *onto* $({\pi a}/{2},[\sin(\pi a)]/[2(1-a)])$.

The following formulas were presented in [2]:

2.5 $$\begin{aligned} &\frac{d\mathscr {K}_{a}(r)}{dr}=\frac{2(1-a)(\mathscr {E}_{a}(r)-r^{\prime 2}\mathscr {K}_{a}(r))}{rr^{\prime 2}},\qquad \frac{d\mathscr {E}_{a}(r)}{dr}= \frac{2(1-a)(\mathscr {K}_{a}(r)-{\mathscr {E}_{a}}(r))}{r}, \end{aligned}$$

2.6 $$\begin{aligned} &\frac{d(\mathscr {E}_{a}(r)-r^{\prime 2}\mathscr {K}_{a}(r))}{dr}=2ar\mathscr {K}_{a}(r), \qquad\frac{d(\mathscr {K}_{a}(r)-\mathscr {E}_{a}(r))}{dr}= \frac{2(1-a)r \mathscr {E}_{a}(r)}{r^{\prime 2}} . \end{aligned}$$

### Lemma 2.2

([2], Lemma 2.3)

*Let* $I\subset\mathbb{R}$ *be an interval*, *and let* $f,g:I\rightarrow (0,\infty)$. *If both* *f*, *g* *are convex and increasing* (*decreasing*), *then the product* $f\cdot g$ *is convex*.

The following lemma follows from Theorem 1.7 in [1].

### Lemma 2.3

*For all* $a,b\in(0,\infty)$, *the function*

2.7 $$\begin{aligned} I(x)=(1-x)^{4}F(a,b;a+b;x) \end{aligned}$$

*is a strictly decreasing automorphism of* $(0,1)$ *if and only if* $4ab\leq a+b $.

### Lemma 2.4

*The function*

2.8 $$\begin{aligned} J(r)=\frac{(1-r^{2}) (\mathscr{E}_{a}(r)-((4a-1)r^{2}+1)\mathscr {K}_{a}(r) )}{4a(1-a)r^{3}} \end{aligned}$$

*is increasing from* $(0,1)$ *onto* $(-\infty,0)$.

### Proof

Let

$$\begin{aligned} f_{1}(x)=(1-x) \bigl(F(a-1,1-a;1;x)-\bigl((4a-1)x+1\bigr)F(a,1-a;1;x) \bigr). \end{aligned}$$

By the series expansion for $F(a,b;c;x)$ we have

2.9 $$\begin{aligned} f_{1}(x) &=(1-x) \Biggl(\sum_{n=0}^{\infty} \frac{(a-1,n)(1-a,n)}{n!}\cdot\frac {x^{n}}{n!}- \bigl((4a-1)x+1 \bigr)\sum _{n=0}^{\infty}\frac {(a,n)(1-a,n)}{n!}\cdot\frac{x^{n}}{n!} \Biggr) \\ &=(1-x) \Biggl(\sum_{n=0}^{\infty} \biggl( \frac{(a-1,n)(1-a,n)}{n!}-\frac {(a,n)(1-a,n)}{n!} \biggr)\cdot\frac{x^{n}}{n!} \\ &\quad {} -\sum _{n=0}^{\infty}\frac{(4a-1)(a,n)(1-a,n)}{n!}\cdot \frac {x^{n+1}}{n!} \Biggr) \\ &=(1-x)\sum_{n=1}^{\infty}\frac {(a,n-1)(1-a,n-1)}{n!n!} \bigl(-4an^{2}+an\bigr)x^{n} \\ &=\sum_{n=1}^{\infty}\frac {(a,n-1)(1-a,n-1)}{n!n!} \bigl(-4an^{2}+an\bigr)x^{n} \\ &\quad {}-\sum _{n=1}^{\infty}\frac {(a,n-1)(1-a,n-1)}{n!n!}\bigl(-4an^{2}+an \bigr)x^{n+1} \\ &=-3ax \\ &\quad {}+\sum_{n=2}^{\infty}\frac{(a,n-2)(1-a,n-2)}{n!n!} an \bigl(\bigl(4n+4a^{2}-4a-6\bigr)n+a+2-a^{2} \bigr)x^{n}. \end{aligned}$$

By the definition of the generalized elliptic integrals of the first and second kinds (1.2) we have

$$\begin{aligned} J(r)&=\frac{\frac{\pi}{2}f_{1}(r^{2})}{4a(1-a)r^{3}} \\ &=\frac{\pi}{8a(1-a)} \Biggl(-\frac{3a}{r}\\ &\quad {}+\sum _{n=2}^{\infty}\frac {(a,n-2)(1-a,n-2)}{n!n!}an \bigl( \bigl(4n+4a^{2}-4a-6\bigr)n+a+2-a^{2} \bigr)r^{2n-3} \Biggr). \end{aligned}$$

Since $0< a<1$, $n\geq2$, we have $(4n+4a^{2}-4a-6)n+a+2-a^{2}>0$, and hence $J(r)$ is an increasing function on $(0,1)$. From this formula it is easy to see that $\lim_{r\rightarrow0^{+}}J(r)=-\infty$. By Lemma 2.3 we have that $\lim_{r\rightarrow1^{-1}}J(r)=0$. □

### Lemma 2.5

([6], Lemma 2.1)

*For* $-\infty< a< b<\infty$, *let* $f,g: [a,b]\rightarrow R$ *be continuous on* $[a, b]$ *and differentiable on* $(a, b)$. *Let* $g'(x) \neq0$ *on* $(a, b)$. *If* $f'(x)/g'(x)$ *is increasing* (*decreasing*) *on* $(a, b)$, *then so are*

$$\begin{aligned} \frac{f(x)-f(a)}{g(x)-g(a)} \quad\textit{and}\quad\frac{f(x)-f(b)}{g(x)-g(b)}. \end{aligned}$$

*If* $f'(x)/g'(x)$ *is strictly monotone*, *then the monotonicity in the conclusion is also strict*.

## Main results and proofs

In this section, we present and prove two main theorems.

### Theorem 3.1

*The function* $f_{a}(r)$ *in* (1.6) *is increasing and convex from* $(0,1)$ *onto* $(\frac{ \pi a(1-a)}{\sin(\pi a)}, [4]\frac{\sin(\pi a)}{\pi a(1-a)} )$. *In particular*,

3.1 $$\begin{aligned} \frac{\pi a(1-a)}{\sin(\pi a)}+\alpha r< f_{a}(r)< \frac{\pi a(1-a)}{\sin (\pi a)}+ \biggl( \frac{\sin(\pi a)}{\pi a(1-a)}-\frac{\pi a(1-a)}{\sin (\pi a)} \biggr)r \end{aligned}$$

*for* $r\in(0,1)$ *with the best constant* $\alpha=0$, $\beta=\frac{\sin(\pi a)}{\pi a(1-a)}-\frac{\pi a(1-a)}{\sin(\pi a)}$. *These two inequalities are sharp as* $r\rightarrow0$, *whereas the second inequality is sharp as* $r\rightarrow1$.

### Proof

Let

$$\begin{aligned} f^{1}_{a}(r)=\frac{\mathscr{E}_{a}(r)-r^{\prime 2}\mathscr{K}_{a}(r)}{r^{2}}. \end{aligned}$$

Then

$$\begin{aligned} f_{a}(r)=\frac{\mathscr{E}_{a}(r)-r^{\prime 2}\mathscr{K}_{a}(r)}{r^{2}}\cdot\frac {r^{\prime 2}}{\mathscr{E'}_{a}(r)-r^{2}\mathscr{K'}_{a}(r)}=f^{1}_{a}(r) \cdot\frac {1}{f^{1}_{a}(r')}. \end{aligned}$$

By Lemma 2.1, $f_{a}^{1}(r)$, $1/f^{1}_{a}(r')$ are positive increasing functions on $(0,1)$, and hence $f_{a}(r)$ is also an increasing function on $(0,1)$. Since $f_{a}^{1}(r)$ is a convex function by Lemma 2.1, the desired convexity of $f_{a}(r)$ will follow from Lemma 2.2 if we prove that $1/f^{1}_{a}(r')$ is a convex function on $(0,1)$.

According to (2.6), we have

$$\begin{aligned} \biggl(\frac{1}{f_{a}^{1}(r)} \biggr)'= \biggl(\frac{r^{2}}{\mathscr {E}_{a}(r)-r^{\prime 2}\mathscr{K}_{a}(r)} \biggr)'=\frac{g_{1}(r)}{g_{2}(r)}, \end{aligned}$$

where

$$\begin{aligned} g_{1}(r)=2 \bigl(\mathscr{E}_{a}(r)-\mathscr{K}_{a}(r)+(1-a)r^{2} \mathscr {K}_{a}(r) \bigr),\qquad g_{2}(r)=\frac{ (\mathscr{E}_{a}(r)-r^{\prime 2}\mathscr{K}_{a}(r) )^{2}}{r}. \end{aligned}$$

Obviously, $g_{1}(0^{+})=0$. By Lemma 2.1 we get $g_{2}(0^{+})=0$. Moreover,

$$\begin{aligned} \frac{g'_{1}(r)}{g'_{2}(r)}=\frac{4a(1-a)r^{3}}{(1-r^{2}) (\mathscr {E}_{a}(r)-((4a-1)r^{2}+1)\mathscr{K}_{a}(r) )}=\frac{1}{J(r)}, \end{aligned}$$

where $J(r)$ is defined by (2.8). Hence, by Lemma 2.4 and Lemma 2.5, $({1}/{f_{a}^{1}(r)} )'$ is decreasing, so that $({1}/{f_{a}^{1}(r')} )'$ is increasing, and ${1}/{f_{a}^{1}(r')}$ is convex on $(0,1)$. □

### Theorem 3.2

*The function* $g_{a}(r)$ *in* (1.7) *is strictly increasing and strictly convex from* $(0,1)$ *onto* $(\frac{\pi a}{2}-\frac{\sin(\pi a)}{2(1-a)},\frac{\sin(\pi a)}{2(1-a)}-\frac{\pi a}{2} )$. *Moreover*, *for all* $r\in(0,1)$, *we have*

3.2 $$\begin{aligned} \frac{\pi a}{2}-\frac{\sin(\pi a)}{2(1-a)}+\alpha r< g_{a}(r)< \frac{\pi a}{2}-\frac{\sin(\pi a)}{2(1-a)}+\beta r \end{aligned}$$

*with the best constants* $\alpha=0$ *and* $\beta=\frac{\sin(\pi a)}{1-a}-\pi a$. *These two inequalities are sharp as* $r\rightarrow0$, *whereas the second inequality is sharp as* $r\rightarrow1$.

### Proof

Let

$$\begin{aligned} M_{a}(r)=\frac{\pi}{2r^{2}} \bigl(F\bigl(a-1,1-a;1;r^{2} \bigr)-r^{\prime 2}F\bigl(a,1-a;1;r^{2}\bigr) \bigr). \end{aligned}$$

By the series expansion for $F(a,b;c;x)$ we obtain

3.3 $$\begin{aligned} M_{a}(r)=\frac{a\pi}{2}F\bigl(a,1-a;2;r^{2} \bigr). \end{aligned}$$

Then

3.4 $$\begin{aligned} g_{a}(r)=M_{a}(r)-M_{a} \bigl(r'\bigr)=\frac{a\pi}{2} \bigl(F\bigl(a,1-a;2;r^{2} \bigr)-F\bigl(a,1-a;2;1-r^{2}\bigr) \bigr). \end{aligned}$$

Using the differentiation formula (2.2), we have

3.5 $$\begin{aligned} g'_{a}(r)&=\frac{a^{2}(1-a)\pi}{2}r \bigl(F \bigl(a+1,2-a;3;r^{2}\bigr)+F\bigl(a+1,2-a;3;1-r^{2}\bigr) \bigr), \end{aligned}$$

3.6 $$\begin{aligned} g''_{a}(r)&=\frac{a^{2}(1-a)\pi}{2} \biggl(F \bigl(a+1,2-a;3;r^{2}\bigr)+F\bigl(a+1,2-a;3;1-r^{2}\bigr) \\ &\quad{}+\frac{2(a+1)(2-a)}{3}r^{2} \bigl(F\bigl(a+2,3-a;4;r^{2} \bigr)-F\bigl(a+2,3-a;4;1-r^{2}\bigr) \bigr) \biggr) . \end{aligned}$$

By formula (2.1),we get

3.7 $$\begin{aligned} F\bigl(a+2,3-a;4;1-r^{2}\bigr)=\frac{1}{r^{2}}F \bigl(a+1,2-a;4;1-r^{2}\bigr). \end{aligned}$$

Using the contiguous relation (2.4), we take $\alpha=a+1$, $\rho=2-a$, $\sigma=3$, and $z=1-r^{2}$ and obtain

$$\begin{aligned} &(a+1)F\bigl(a+1,2-a;4;1-r^{2}\bigr)\\ &\quad =3F\bigl(a+1,2-a;3;1-r^{2} \bigr)-(2-a)F\bigl(a+1,3-a;4;1-r^{2}\bigr). \end{aligned}$$

Hence, it follows from (3.6), (3.7), and the last formula that

$$\begin{aligned} &\frac{2}{a^{2}(1-a)\pi }g''_{a}(r) \\ &\quad =F \bigl(a+1,2-a;3;r^{2}\bigr)+F\bigl(a+1,2-a;3;1-r^{2}\bigr) \\ &\qquad{}+\frac{2(a+1)(2-a)}{3}r^{2} \biggl(F\bigl(a+2,3-a;4;r^{2} \bigr)-\frac {F(a+2,3-a;4;1-r^{2})}{r^{2}} \biggr) \\ &\quad =F\bigl(a+1,2-a;3;r^{2}\bigr)+\frac {2(a+1)(2-a)r^{2}}{3}F \bigl(a+2,3-a;4;r^{2}\bigr) \\ &\qquad{}+(2a-3)F\bigl(a+1,2-a;3;1-r^{2}\bigr)+\frac {2(2-a)^{2}}{3}F \bigl(a+1,3-a;4;1-r^{2}\bigr) \\ &\quad >1+(2a-3)F\bigl(a+1,2-a;3;1-r^{2}\bigr)+\frac {2(2-a)^{2}}{3}F \bigl(a+1,3-a;4;1-r^{2}\bigr). \end{aligned}$$

By the series expansion for $F(a,b;c;x)$ we have

3.8 $$\begin{aligned} &(2a-3)F\bigl(a+1,2-a;3;1-r^{2}\bigr)+\frac {2(2-a)^{2}}{3}F \bigl(a+1,3-a;4;1-r^{2}\bigr) \\ &\quad =\sum_{n=0}^{\infty} \biggl(\frac {2(a+1,n)(3-a,n)(2-a)^{2}}{(3,n+1)}+ \frac {(a+1,n)(2-a,n)(2a-3)}{(3,n)} \biggr)\frac{(1-r^{2})^{n}}{n!} \\ &\quad =\sum_{n=0}^{\infty}\frac{(2-a,n)(n+2a^{2}-2a-1)(a+1,n)}{(3,n+1)} \frac {(1-r^{2})^{n}}{n!} \\ &\quad>\frac{2a^{2}-2a-1}{3}+\frac{a(2-a)(a^{2}-1)}{6}\bigl(1-r^{2}\bigr). \end{aligned}$$

Hence

3.9 $$\begin{aligned} \frac{2}{a^{2}(1-a)\pi}g''_{a}(r)>1+ \frac{2a^{2}-2a-1}{3}+\frac {a(2-a)(a^{2}-1)}{6}\bigl(1-r^{2}\bigr). \end{aligned}$$

Through direct calculation we have

3.10 $$\begin{aligned} &1+\frac{2a^{2}-2a-1}{3}+\frac{a(2-a)(a^{2}-1)}{6} \\ &\quad =\frac {-a^{4}+2a^{3}+5a^{2}-6a+4}{6}>0 ,\quad \forall a \in(0,1). \end{aligned}$$

Then we get $g''_{a}(r)>0$. Thus $g_{a}(r)$ is strictly convex on $(0,1)$. According to (3.3) and (2.3), we have

3.11 $$\begin{aligned} M_{a}(0)=\frac{a\pi}{2},\qquad M'_{a}(0)=0, \qquad\lim_{r\rightarrow1^{-}}M_{a}(r)=\frac{\sin(\pi a)}{2(1-a)}. \end{aligned}$$

Applying Lemma 2.3 and (2.6), we have

$$\begin{aligned} g'_{a}(0)=\lim_{r\rightarrow0} \frac{M_{a}(r)-M_{a}(0)}{r}-\frac {M_{a}(r')-M_{a}(1)}{r} =\lim_{x\rightarrow1} \frac{x'}{x^{4}} \bigl(\bigl(2-x^{2}\bigr)\mathscr {K}_{a}(x)-2\mathscr {E}_{a}(x) \bigr)=0 . \end{aligned}$$

Because of $g''_{a}(r)>0$, $g'_{a}(r)$ is increasing on $(0,1)$, and $g'_{a}(0)=0$. Then the monotonicity of $g_{a}(r)$ on $(0,1)$ is obtained. It follows from the convexity of $g_{a}(r)$ that, for $x\in(0, 1)$,

3.12 $$\begin{aligned} \frac{\pi a}{2}-\frac{\sin(\pi a)}{2(1-a)}< g_{a}(r)< \frac{\pi a}{2}- \frac{\sin(\pi a)}{2(1-a)}+ \biggl(\frac{\sin(\pi a)}{1-a}-\pi a \biggr)r. \end{aligned}$$

 □

### Corollary 3.3

*Let*

3.13 $$\begin{aligned} L_{a}(p,q)=g_{a}(pq)-g_{a}(p)-g_{a}(q). \end{aligned}$$

*Then we have*

3.14 $$\begin{aligned} \frac{\pi a}{2}-\frac{\sin(\pi a)}{2(1-a)}< L_{a}(p,q)< \frac{\sin(\pi a)}{2(1-a)}- \frac{a\pi}{2} \end{aligned}$$

*for all* $p,q\in(0,1)$.

### Proof

By direct calculation we obtain

$$\begin{aligned} \frac{\partial}{\partial p}L_{a}(p,q)=sg'_{a}(pq)-g'_{a}(p), \qquad \frac{\partial^{2}}{\partial p\,\partial q}L_{a}(p,q)=g'_{a}(pq)+pqg''_{a}(pq). \end{aligned}$$

Considering the positivity of $g'_{a}$ and $g''_{a}$ on $(0,1)$, we have

$$\begin{aligned} \frac{\partial^{2}}{\partial p\,\partial q}L_{a}(p,q)>0, \end{aligned}$$

This means that $\frac{\partial}{\partial p}L_{a}(p,q)$ is strictly increasing with respect to *q*. So we have

3.15 $$\begin{aligned} \frac{\partial}{\partial p}L_{a}(p,q)< \frac{\partial}{\partial p}L_{a}(p,q)\bigg|_{q=1}=0. \end{aligned}$$

Then the monotonicity of $L_{a}(p,q)$ with respect to *p* is obtained, which leads to

$$\begin{aligned} \frac{\pi a}{2}-\frac{\sin(\pi a)}{2(1-a)}< L_{a}(p,q)< \frac{\sin(\pi a)}{2(1-a)}- \frac{a\pi}{2}. \end{aligned}$$

 □

### Remark 3.4

Taking $a= 1/2$ in Theorems 3.1 and 3.2, we get Theorems 1.1 and 1.2.
